# E3 Ubiquitin Ligase in Anticancer Drugdsla Resistance: Recent Advances and Future Potential

**DOI:** 10.3389/fphar.2021.645864

**Published:** 2021-04-15

**Authors:** Yuanqi Liu, Chaojun Duan, Chunfang Zhang

**Affiliations:** ^1^ Department of Thoracic Surgery, Xiangya Hospital, Central South University, Changsha, China; ^2^ Hunan Engineering Research Center for Pulmonary Nodules Precise Diagnosis & Treatment, Changsha, China; ^3^ Department of Oncology, Xiangya Hospital, Central South University, Changsha, China; ^4^ National Clinical Research Center for Geriatric Disorders, Changsha, China

**Keywords:** E3 ubiquitin ligase, cancer, cancer treatment, drugresistance, drug

## Abstract

Drug therapy is the primary treatment for patients with advanced cancer. The use of anticancer drugs will inevitably lead to drug resistance, which manifests as tumor recurrence. Overcoming chemoresistance may enable cancer patients to have better therapeutic effects. However, the mechanisms underlying drug resistance are poorly understood. E3 ubiquitin ligases (E3s) are a large class of proteins, and there are over 800 putative functional E3s. E3s play a crucial role in substrate recognition and catalyze the final step of ubiquitin transfer to specific substrate proteins. The diversity of the set of substrates contributes to the diverse functions of E3s, indicating that E3s could be desirable drug targets. The E3s MDM2, FBWX7, and SKP2 have been well studied and have shown a relationship with drug resistance. Strategies targeting E3s to combat drug resistance include interfering with their activators, degrading the E3s themselves and influencing the interaction between E3s and their substrates. Research on E3s has led to the discovery of possible therapeutic methods to overcome the challenging clinical situation imposed by drug resistance. In this article, we summarize the role of E3s in cancer drug resistance from the perspective of drug class.

## Introduction

Cancer is a multifactorial disease and is considered the most severe public health issue worldwide (Siegel et al., 2019). Drug therapy is the main treatment for patients with advanced cancer. The drugs currently used for tumor treatment include platinum drugs, antitumor antibiotics, alkylating agents, hormones, molecular targeting agents, and immunotherapy. The use of tumor drugs will inevitably lead to drug resistance, which manifests as tumor recurrence (Glickman and Sawyers, 2012; Vasan et al., 2019).

Several mechanisms have been found to underlie anticancer drug resistance, including the effects of cancer stem cells (CSCs), epithelial-mesenchymal transition (EMT), and DNA damage repair (DDR) (Gong et al., 2018). Identifying the key molecules in these processes can help us understand the occurrence of drug resistance, and these key molecules play an essential role in predicting and reversing resistance to anticancer drugs. However, the mechanisms have not yet been elucidated.

### Ubiquitination and E3 Ubiquitin Ligases

Protein ubiquitination-based modification can regulate various signal-mediated cell death responses and plays an essential role in the occurrence, development, and outcome of cancer (Nalepa et al., 2006). Ubiquitination refers to the process by which ubiquitin is covalently bound to target proteins under the catalysis of a series of enzymes. The ubiquitination process usually requires the cooperation of the E1 ubiquitin-activating enzymes (E1s), E2 ubiquitin-coupling enzymes (E2s), and E3 ubiquitin ligases (E3s) (Buetow and Huang, 2016). Mechanistically, ubiquitin is activated in an ATP-dependent manner, inducing a thioester bond with an E1. The moieties are then transferred to the active site cysteine of the E2. The E3 binds to both the E2∼Ub thioester and the substrate, catalyzing the transfer of ubiquitin from the active site cysteine of the E2 to the substrate lysine or N terminus (Tatham et al., 2013; Berndsen and Wolberger, 2014).

Ubiquitin chain topology determines the fate of ubiquitylated proteins (Ikeda and Dikic, 2008). Usually, ubiquitin-dependent proteolysis is associated with K48-linked and K11-linked ubiquitin chains. However, activation of signaling pathways is mainly dependent on K63‐linked or M1-linked ubiquitin chains. Ubiquitin-mediated proteolysis is essential for the maintenance of protein homeostasis because it removes misfolded or unwanted proteins. The non-proteasomal ubiquitin-mediated assembly of signaling complexes also plays a pivotal role in several cellular processes, such as autophagy, DNA repair, and endocytosis (Bennett and Harper, 2008; Chen and Sun, 2009; Cappadocia and Lima, 2018). Given these phenomena, it is understandable that the dysregulation of ubiquitination can lead to genetic and epigenetic alterations in cancer.

**FIGURE 1:**
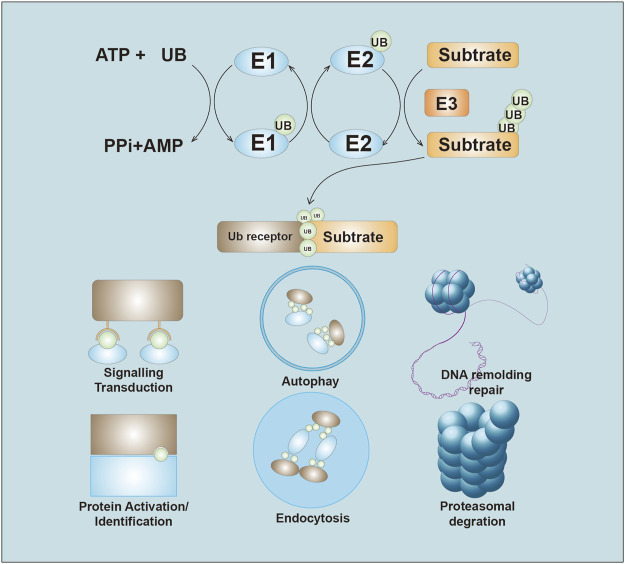
Overview of the Ubiquitin system. Ubiquitin is initiate through the thioester bond with E1 in the ATP-depend manner. The activated ubiquitin then be transferred to the E2. E3 ligases recognize and transfer the ubiquitin to substrate. The ubiquitin chains lead to several ending, including proteasome-mediated degradation, ubiquitination signals transduction, autophagy, DNA remolding and repair, Protein identification, and endocytosis.

E3s are critical components in the ubiquitination reaction owing to their strict control of both substrate affinity and specificity (Zheng and Shabek, 2017). E3s are a large class of proteins, and there are over 800 putative functional E3s (Ciechanover, 2015). E3s have been classified into three subgroups: E3s containing really interesting new gene (RING) and UFD2 homology (U-box) domains, the 28-member homologous to E6AP carboxyl terminus (HECT) E3 family, and the 14‐member RING‐between‐RING (RBR) E3 family (Uchida and Kitagawa, 2016). The diversity of the E3 substrates contributes to the diverse functions of E3s (Li et al., 2014), and as a result, E3s are closely related to tumorigenesis because they regulate oncogenes and tumor suppressors (Senft et al., 2018). In addition, the substrate specificity of E3s suggests that they have promise as anticancer drug targets (Wang et al., 2017). Here, for the first time, we summarize the role of E3s in anticancer drug resistance from the perspective of drug class (Figure 1).

### Platinum Drugs

Platinum drugs are widely used in the treatment of human cancers. The zinc-finger E3s MUL1 and UBR5 was found to be involved in platinum resistance. The E3 UBR5 was amplified and overexpressed in ovarian cancer (OC). Higher UBR5 expression was observed in platinum-resistant OC tumor tissue than in normal tissues. Overexpression of UBR5 induced cisplatin resistance in OC cells both *in vivo* and *in vitro*. Moreover, UBR5 knockdown via siRNA partly reversed platinum resistance in OC cells (O'Brien et al., 2008; Bradley et al., 2014). Mitochondrial E3 ubiquitin-protein ligase 1 (MUL1) is an E3 that interacts with and negatively regulates AKT. The degradation of AKT was found to lead to cisplatin sensitivity in OC cells (Lee et al., 2019). A decrease in EMT in cisplatin-resistant nasopharyngeal carcinoma (NPC) cells was observed after upregulation of NEDD4 in cells, suggesting that NEDD4 could be a novel therapeutic target for overcoming drug resistance in NPC (Feng et al., 2017).

Tripartite motif (TRIM) family proteins, most of which have E3 activities, control important cellular processes such as intracellular signaling, innate immunity, transcription, autophagy, and carcinogenesis (Hatakeyama, 2017). TRIM25 expression was identified to be significantly lower in the cisplatin-resistant non-small cell lung carcer (NSCLC) cell line A549 than in control cell lines (Qin et al., 2012). Overexpression of TRIM32 promoted degradation of Abi2, resulting in enhancement of cell growth, transforming activity, and cell motility. Moreover, TRIM32 suppressed the apoptosis induced by cisplatin in the hepatocellular carcinoma (HCC) cell line HEp2. Overexpression of TRIM32 in the HCC cell line also induced resistance to another platinum drug, oxaliplatin (Cui et al., 2016). Increased TRIM11 expression inhibits the apoptosis induced by cisplatin, and TRIM11 functions as an oncogene related to drug resistance both *in vivo* and *in vitro*. TRIM11 destabilized Daple in a p62-selective autophagic manner, further upregulating β-catenin expression to induce enhanced expression of ABCC9, which can transport chemotherapeutic drugs (Zhang et al., 2020). Autophagy can be a key mechanism of resistance to chemotherapy (Onorati et al., 2018). TRIM65 was found to be upregulated in NSCLC, and its overexpression promoted NSCLC cell resistance to cisplatin (Li et al., 2016). The inhibition of miR-138–5p attenuated the effects of TRIM65 knockdown on autophagy and cisplatin-induced apoptosis, suggesting that TRIM65 regulates cisplatin resistance in NSCLC by regulating miR-138–5p (Pan et al., 2019). TRIM59 was also found to be overexpressed in cisplatin-resistant A549 cells, and its overexpression in these cells resulted in increased cisplatin resistance. TRIM59 enhanced the ubiquitination of PTEN, a critical upstream regulator of HK2. The regulation of the PTEN/AKT/HK2 pathway induced by TRIM59 might provide insights into overcoming cancer resistance to cisplatin treatment (He and Liu, 2020).

Another type of RING-box-containing E3s, RING finger proteins (RNFs), play a role in platinum resistance. RNF38 was proposed as a biomarker of poor NSCLC prognosis, and its silencing increased the sensitivity of NSCLC cells to cisplatin (Wu et al., 2020). RNF138 was more highly expressed in cisplatin-resistant gastric cancer (GC) cell lines than in normal cell lines and modulated cisplatin resistance in these GC cells (Lu et al., 2018). Pellino family proteins (Pellino-1, 2, and 3) are E3s that contain C-terminal RING-like domains. Pellino-1 overexpression conferred NSCLC cells with resistance to the apoptosis induced by cisplatin or paclitaxel (Jeon et al., 2016).

The F-box-containing family member FBXW7 is one of the four subunits of the SKP1-cullin-F-box (SCF)-E3 complex, which functions in phosphorylation-dependent ubiquitination (Skaar et al., 2014). In NSCLC, FBXW7 upregulation significantly increased cisplatin chemosensitivity and abrogated the mesenchymal phenotype of NSCLC cell lines (Yu et al., 2013). Another report by Guodong X et al. indicated that FBXW7 could interact with Snai1 in NSCLC cell lines and directly degrade its expression, resulting in suppression of cisplatin and sorafenib resistance (Guodong et al., 2018). In colorectal carcinoma (CRC) cells, FBXW7 deficiency induced by mutation or loss can lead to the aberrant phosphorylation of p53 at serine 15 and further promote resistance to oxaliplatin. An understanding of the regulation of phospho-p53 (Ser15) by FBXW7 E3 activity could provide important clues for the clinical targetability of this axis (Perez-Losada et al., 2005; Li et al., 2015). In NPC, upregulation of FBXW7 significantly increased cisplatin-based chemosensitivity (Song et al., 2015). The CRL4 expression level was increased in cisplatin-resistant OC cells. CRL4 knockdown with shRNAs was able to reverse the cisplatin resistance of OC cells (Hu et al., 2019). In CRC, knockdown of CUL4A sensitized parental CRC cells to cisplatin (Englinger et al., 2017). CUL4B destabilized HP1α, a gene that suppresses the open confirmation of chromatin that is important for the DDR and DNA repair. The DDR and DNA repair are believed to be reasons for cisplatin resistance (Kim et al., 2017).

F-box-only proteins are the substrate-recognition component of the SCF-E3 complex. The relatively low level of FBXO22 in A549 cells contributes to an accumulation of CD147 and the cisplatin resistance of the cells (Wu et al., 2017). Additionally, FBXO21 was found to ubiquitinate and destabilized P-glycoprotein (P-gp), resulting in attenuation of multidrug resistance. However, a stem cell marker, CD44, was found to inhibit FBXO21-directed degradation of P-gp and promote multidrug resistance (Ravindranath et al., 2015). Using a microarray, another E3, FBXO32, was newly identified as a negative regulator of EMT in urothelial carcinoma (UC) tumors after they had acquired platinum resistance. FBXO32 dysregulation in platinum-resistant UC cells resulted in elevated expression of the EMT marker snail and decreased expression of E-cadherin (Tanaka et al., 2016).

SKP2, also known as F-box and leucine-rich repeat protein (FBXL1), is a member of the FBXL subfamily of F-box proteins and plays a pivotal role in cell cycle progression and proliferation. Evidence has shown that SKP2 can interact with Akt and facilitate its ubiquitination. K63-mediated AKT ubiquitination can be mediated by Skp2 and can regulate NPC to induce cisplatin resistance (Yu et al., 2019). Overexpression of SKP2 reduced the expression of p27Kip1, cyclin E, and p21Cip1, increased the proportion of S-phase cells, and increased resistance against cisplatin in NSCLC cell lines (Ishii et al., 2004).

E3s targeting P53 play a role in cisplatin resistance. MDM2, an important regulator of P53, controls cisplatin resistance in multiple cancers. MDM2 interacted and destabilized P53 and induced tumor cell resistance to cisplatin (Muscolini et al., 2011; Sheng et al., 2017). The genes encoding molecules that interfere with the interaction between MDM2 and p53 might also lead to drug resistance. Some genes, such as zinc-finger CCHC-type containing 10 (ZCCHC10) and NUMB, inhibit cisplatin resistance by interfering with P53 ubiquitination mediated by MDM2 (Colaluca et al., 2008; Ning et al., 2019). TRAF6, an E3 that controls p53 mitochondrial translocation, was found to be overexpressed in CRC tissues. TRAF6 overexpression negatively correlates with apoptosis and predicts poor response to cisplatin-based chemotherapy and radiotherapy (Zhang et al., 2016). Cisplatin can enhance the FLIP-p53-Itch interaction, inducing FLIP ubiquitination and degradation in a p53-and Itch-dependent manner. These results suggest that the modulation of FLIP content may be an effective strategy for overcoming chemoresistance in OC (Abedini et al., 2008; Abedini et al., 2010).

**FIGURE 2:**
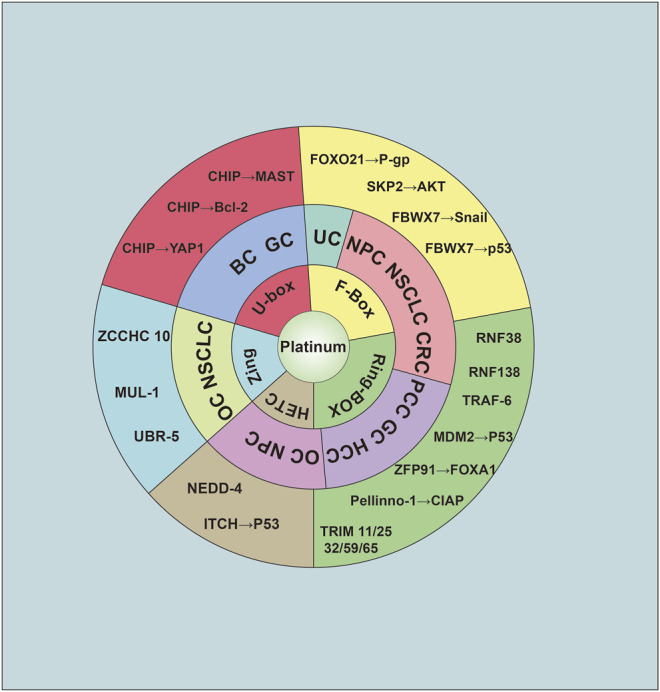
The relationship of E3 ligase and platinum resistance. The E3 was cataloged as five group: U-box, F-box, Ring-Box, HETC, and Zing finger. Abbreviation was as follow: BC: Breast cancer; GC: Gastric cancer; UC, urothelial carcinoma; NPC, nasopharyngeal carcinoma; NSCLC, non-small cell lung cancer; CRC, colorectal carcinoma; PCC, pancreatic carcinoma; HCC, hepatocellular carcinoma; OC, ovarian cancer.

The U-box domain-containing E3 CHIP has been studied to help understand drug sensitivity. CHIP knockdown increases the proportion of cisplatin-sensitive cells. CHIP can act as an activator of Bcl-2 expression levels to suppress breast cancer (BC) malignant progression (Tsuchiya et al., 2015). In addition to CHIP’s role in BC, Dong‐E Tang et al. revealed that CHIP could ubiquitinate YAP1 at the K280 site by K48‐linked polyubiquitination and also generated a human GC cell line with resistance to cisplatin resistance (Tang et al., 2019). Microtubule-associated serine/threonine kinase 1 (MAST1) mediates cisplatin resistance in human cancers. Pan C et al. used a proteomics screen to identify CHIP-destabilized MAST1. The MAST1 destabilization resulted in hsp90B-induced sensitivity to cisplatin (Pan et al., 2019). ZFP91 knockdown reduced FOXA1 polyubiquitination, which decreased FOXA1 turnover and enhanced cellular sensitivity to cisplatin therapy (Tang et al., 2020). E3s and their corresponding substrates are summarized in Figure 2.

### Drugs Derived From Plants

Paclitaxel is an M-phase-specific plant drug initially derived from the Pacific yew and has become the first member of the taxane family to be used in cancer chemotherapy (Bernabeu et al., 2017). In a single-cell RNA sequencing analysis, protein ubiquitination was identified as the most differentially regulated pathway in docetaxel-resistant prostate cancer (PC) cells (Schnepp et al., 2020).

In NSCLC, BC and GC, silencing FBXW7 resulted in enhanced Taxol resistance (Yokobori et al., 2014; Gasca et al., 2016). However, in dormant BC cells, disruption of FBXW7 resulted in a shift of tumor cells from the quiescent state, rendering them susceptible to chemotherapy (Shimizu et al., 2019). miR-363 expression can promote chemoresistance by directly targeting FBXW7 (Zhang et al., 2016). Another Fox-containing E3, FBXW11, was also found to be involved in the development of Taxol resistance via associations with FOXO3a (Su et al., 2011). In PC, Skp2 silencing or using Skp2 inhibitors restored paclitaxel sensitivity in paclitaxel-resistant PC cells (Yang et al., 2016). EDD, an E3, promotes docetaxel resistance in hormone-refractory PC by regulating Wnt/β-Catenin signaling (Bian et al., 2020). In PC, SPOP knockout was found to confer resistance to cell death caused by docetaxel (Shi et al., 2019). Taxol decreased the expression of HDAC3 while increasing the expression of SIAH2 in melanoma cells. In addition, the E3 ligase SIAH2 can interact with HDAC3 and by so doing confers resistance to Taxol (Kim et al., 2015).

E3s targeting p53 also play roles in Taxol resistance. Pirh2, a RING finger-containing E3, can lead to polyubiquitination and proteasomal degradation of p53. The ectopic expression of Pirh2 enhanced cell proliferation, resistance to doxorubicin, and migration potential (Daks et al., 2016). The protease HAUSP is a critical component of the p53-Mdm2 pathway and acts as a specific deubiquitinase for p53 and Mdm2 and is thus essential for p53 regulation. HAUSP downregulation causes resistance to another plant-derived drug, camptothecin, and camptothecin-induced apoptosis (Becker et al., 2008). Parkin interferes with paclitaxel-induced microtubule assembly and stabilization by directly binding the microtubules on the outer cell surface. In addition, Parkin promotes the activity of paclitaxel to trigger multinucleation and apoptosis. Moreover, clinical data have revealed that the response of patients to preoperative paclitaxel therapy is correlated with Parkin expression (Wang et al., 2009).

### Antimetabolite Drugs

Fludarabine is a DNA synthesis inhibitor (Lukenbill and Kalaycio, 2013). *In vitro* and *in vivo* experiments showed that COP1 overexpression reduced HG3 cell sensitivity to fludarabine treatment by promoting ubiquitin-dependent p53 degradation (Fu et al., 2018). This result indicates that E3s promoting P53 degradation can also be related to fludarabine resistance.

5-Fluorouracil (5-FU) inhibits thymidylate synthase from activating thymine-induced cell death and functions mainly as an S-phase antimetabolite (Longley et al., 2003). In BC, high preoperative expression of Skp2 was found to be associated with resistance to 5-FU therapy in 94% of patients (Davidovich et al., 2008). Cbl was decreased in 60% of human pancreatic ductal adenocarcinoma (PDAC) cases. Cbl knockdown increased PDAC resistance to gemcitabine and 5-FU (Kadera et al., 2015). TRIM47 is commonly overexpressed and related to poor prognosis in CRC patients. TRIM47 increases the ubiquitination and degradation of SMAD4. The overexpression of TRIM47 elevated CRC chemoresistance in response to 5-FU therapy (Liang et al., 2019). In CRC, high FBXW7 expression downregulated CRY2 through proteasomal degradation and increased CRC cell sensitivity to 5-FU (Fang et al., 2015). Cancer-associated fibroblasts (CAFs) play a pivotal role in creating the tumor microenvironment, which impacts adaptive resistance to chemotherapy (Kalluri, 2016). CRC cells cocultured with CAFs showed increased expression of RANBP2-type and C3HC4-type zinc-finger-containing 1 (RBCK1). Additionally, overexpression of RBCK1 was demonstrated in chemoresistant CRC tumors and CRC patients with poor prognosis. Exogenous expression of RBCK1 or RBCK1 inhibition was able to significantly influence 5-FU sensitivity in CRC cells (Liu et al., 2019).

Gemcitabine is another widely used S-phase antimetabolite drug (Mini et al., 2006). In pancreatic cancer (PCC) cells, FBW7 promoted gemcitabine sensitivity via upregulation of equilibrative nucleoside transporter 1 (ENT1) through lysosome inhibition (Hu et al., 2017). SMURF2 was downregulated in PCC tissues, and its expression was negatively associated with gemcitabine resistance. Upregulation of miR-15b was associated with degradation of SMURF2, and its expression was associated with EMT (Zhang et al., 2015). TRIM31 overexpression conferred gemcitabine resistance to PCC cells by promoting K63-linked polyubiquitination of tumor necrosis factor receptor-associated factor 2 (TRAF2) and sustained the activation of nuclear transcription factor kappa B (NF-κB) in PCC cells (Yu et al., 2018). Cul4A degraded TGFβ1, and its overexpression promoted resistance to gemcitabine in lung cancer. *In vivo* experiments, Cul4A-RNAi combined with gemcitabine chemotherapy inhibited lung cancer tumor growth, suggesting that this combination may provide a new approach for lung cancer treatment (Hung et al., 2015).

Asparaginase is a drug that selectively inhibits tumor cells by hydrolyzing asparagine (Asselin and Rizzari, 2015). FBXW7 overexpression can rescue Wnt-induced sensitization to asparaginase in FBXW7 mutant or wild-type leukaemias. In contrast, the FBXW7 R465C mutant, which has impaired binding to its canonical phosphodegron, abrogated this effect (Hinze et al., 2019).

### Alkylating Agents

Among various antitumour drugs, alkylating agents may be the most widely used category (Bhatt et al., 2017). Alkylating agents are cytotoxic drugs that combine with cell proteins and nucleic acids to kill tumor cells and have a direct toxic effect on cells (Lajous et al., 2019). Temozolomide (TMZ) is a DNA alkylating agent that can penetrate the blood-brain barrier. HERC3, an E3, promotes the ubiquitination-mediated degradation of SMAD7 and consequently activates the TGF-β pathway. Moreover, ectopic HERC3 expression was correlated with TMZ resistance in glioblastoma (GBM) cells (Li et al., 2019). Nucleolin (NCL) is overexpressed in GBM, and its overexpression was found to be positively relative to response to TMZ in GBM cells. The loss of MDM2-mediated NCL ubiquitination resulted in the inhibition of HDAC activity and sensitized GBM cells to TMZ (Ko et al., 2018).

### Anticancer Antibiotics

Doxorubicin is a cytotoxic anthracycline antibiotic that is often used as a tumor chemotherapy agent. Using mass spectrometry analysis, Kamran M et al. found that AURKA restricted FBXL7-induced survivin ubiquitination and degradation in GC, resulting in the promotion of doxorubicin resistance (Kamran et al., 2017). Doxorubicin-resistant HCC cells showed decreased expression of FBXW7. HSF1 was found to play an essential role in transcriptional activation of MDR1 via FBXW7-mediated degradation (Mun et al., 2020). Expression of P-gp on cancer cell surfaces is a critical determinant of anticancer drug resistance (Lin and Yamazaki, 2003). In other words, the reversal of drug resistance can be achieved by modulating the ubiquitination of P-gp (Zhang et al., 2004). Via mass spectrometry analyses, FBXO15/Fbx15 was found to interact with P-gp (Katayama et al., 2013). The downregulation of P-gp expression by UBE2R1-and FBXO15-mediated ubiquitination boosted sensitivity to vincristine and doxorubicin (Katayama et al., 2016).

Zeb1, an influential EMT-related transcription factor, mediated cell resistance to doxorubicin treatment. In HCC doxorubicin-resistant cells, the downregulation of SIAH1 mediated the stability of Zeb1, aiding resensitization of cells to doxorubicin treatment (Long et al., 2019). RNF8 activated K63 ubiquitination of Twist, which induced its translocation to the nucleus for subsequent EMT and CSC functions, thereby conferring doxorubicin resistance (Lee et al., 2016). SMO stabilizes and activates TRAF6, suggesting that the SMO/TRAF6 axis can contribute to doxorubicin resistance in lymphoma (Qu et al., 2018).

Dysregulated cholesterol metabolism in cancer cells may lead to drug resistance. Lower expression of the E3 Trc8 produced a decreased ubiquitination rate of 3-hydroxy-3-methylglutaryl-coenzyme A reductase (HMGCoAR), increased cholesterol synthesis, and increased cholesterol content in multidrug-resistant cells (Gelsomino et al., 2013). Overexpression of the E3 ubiquitin-protein ligase ZNRF2 improved cell survival in the presence of doxorubicin (Xiao et al., 2017). TRIM25 regulated p53 expression in NSCLC tissues and cell lines. Using TRIM25 RNAi increased the doxorubicin sensitivity of lung cancer cell lines (Qin et al., 2016). CUL2 knockdown enhanced cell sensitivity to doxorubicin treatment by regulating MAF1-mediated actin stress fiber integrity and apoptosis (Wang et al., 2019). MDM2, an E3 targeting p53 for degradation, can influence PC and BC cell sensitivity to doxorubicin (Lang et al., 2017; Cheteh et al., 2020). FKBP12 attenuated the cell toxicity of doxorubicin by binding to and degrading MDM2, disrupting the MDM2/MDM4 interaction, and inducing MDM2 self-ubiquitination (Liu et al., 2017). Cbl-b was found to be poorly expressed in multidrug-resistant GC and GC cells. In addition, Cbl was also found to induce cell resistance to adriamycin (Xu et al., 2017; X CHe et al., 2017; Zhang et al., 2015).

### Endocrine Drugs

Endocrine therapy is an optional treatment for patients with hormone sensitivity, especially in PC and BC (Heinlein and Chang, 2004; Waks and Winer, 2019). Androgenic drugs such as abiraterone and enzalutamide can control the progression of PC.

Persistent androgen receptor (AR) activation leads to the loss of efficacy of anti-AR drugs in advanced PC. Reversal of this aberrant activation could be an ideal method for overcoming drug resistance. STUB1 disassociates AR/AR-V7 from HSP70, leading to AR/AR-V7 ubiquitination and degradation, which confers enzalutamide and abiraterone resistance (Liu et al., 2018). Inhibition of protein degradation by blocking Cullin-RING E3 complexes can interfere with the AR–ERG interaction, which is related to survival in PC (Rulina et al., 2016). AMFR can mediate the loss of 11b-hydroxysteroid dehydrogenase-2 (11b-HSD2), which inactivates cortisol, sustaining tumor cortisol concentrations to stimulate enzalutamide resistance. Reinstatement of 11b-HSD2 expression, or AMFR loss, reverses enzalutamide resistance in mouse xenograft tumors (Li et al., 2017).

Tamoxifen, a blocker of estrogen in breast cells, remains a cornerstone in the treatment of BC patients with estrogen receptor-positive tumors (Jordan, 2003). The RING finger protein TRIM2 is highly expressed in tamoxifen-resistant MCF-7 cells. TRIM2 was overexpressed in tamoxifen-resistant BC cells, which led to a reduction in Bim (Yin et al., 2017). The E3 HRD1 was downregulated in tamoxifen-resistant BC cells, and its knockdown significantly increased the survival of MCF7 cells treated with tamoxifen (Wang et al., 2017). The E3 RBCK1 regulated FKBPL stability at the posttranslational level via ubiquitination, and its downregulation increased sensitivity to tamoxifen treatment (Donley et al., 2014). The FBXW2-mediated downregulation of Sox2, a transcription factor conferring drug resistance, suppressed stem cell properties and overcame BC cell resistance to tamoxifen (Yin et al., 2019). The ubiquitin ligase c-Cbl was upregulated during tamoxifen-induced apoptosis of MCF-7 cells. Overexpression of c-Cbl significantly downregulated c-Src protein levels and tamoxifen-induced AKT activity (Yan et al., 2011). In addition, SIAH2 expression is significantly correlated with ER positivity in BC. SIAH2 sensitizes cells to tamoxifen through regulation of ER-a expression (Interiano et al., 2014).

### Targeted Drugs

The fusion of BCR (located on chromosome 22q11.2) and ABL1 (located on chromosome 9q34) leads to chronic myeloid leukemia (CML) (Baccarani et al., 2019). Imatinib is a tyrosine kinase inhibitor that can selectively inhibit BCR/ABL kinase activity and function as an effective therapy for CML. Smith PG et al. found that TGFβ played a key role in imatinib resistance by directly affecting c-Cbl-dependent Lyn ubiquitination and turnover, which resulted in bursts of Lyn kinase activity (Smith et al., 2012). TRAF6, an E3, facilitates the K63 ubiquitination of ULK1, resulting in reversal of imatinib resistance in CML cells (Han et al., 2019). LZTR1 acts as the regulator of RAS ubiquitination and MAPK pathway activation. Johannels W et al. reported that loss of LZTR1 expression could induce resistance to imatinib and rebastinib in CML cell lines (Bigenzahn et al., 2018).

Bortezomib is an effective proteasome inhibitor for cancer treatment that reversibly and selectively inhibits the 20S proteasome (Scott et al., 2016). Using an RNA microarray, researchers found that genes related to ubiquitination were differentially regulated in a bortezomib-resistant cell line (Park et al., 2014). NEDD4-1 ubiquitinates Akt and targets pAkt-Ser473 for proteasomal degradation. Low NEDD4-1 expression has been linked to poor prognosis in patients with multiple myeloma (MM), and NEDD4-1 knockdown results in bortezomib resistance *in vitro* and *in vivo* (Huang et al., 2020). Additionally, BAP1 depletion resulted in decreased gallbladder carcinoma (GBC) sensitivity to bortezomib (Hirosawa et al., 2018). Shen Y et al. demonstrated that silencing DTX3L improved the sensitivity to bortezomib in MM cell lines and increased the expression of apoptosis biomarkers (Shen et al., 2017). Malek E et al. observed increased CUL1 and SKP2 mRNA levels in patient CD138 + cells. Skp2 binds to Cullin-1 and Commd1 and synergistically enhances bortezomib-induced apoptosis (Malek et al., 2017).

Targeting HER2 with an inhibitor can be a treatment strategy for HER-2-positive BC or GC. c-Cbl and CHIP can interact and ubiquitinate HER2, which can be an effective strategy for combatting lapatinib resistance in HER2-positive cancer (Nunes et al., 2016; Huang et al., 2020). In addition, Skp2 silencing sensitized Her2-overexpressing tumors to trastuzumab treatment (Chan et al., 2012). MDM2 inhibition overcame lapatinib resistance in cells with either wild-type or mutant p53 and xenograft models, suggesting the potential of therapy directed against MDM2 for overcoming lapatinib resistance (Kurokawa et al., 2013). In GC, miR-223, which can regulate FBXW7, decreased GC cell sensitivity to trastuzumab (Eto et al., 2015). Jagged-1-mediated activation of Notch-1 can lead to trastuzumab resistance. The E3 Mindbomb-1 was required for Jagged-1 ubiquitination and subsequent Notch activation, which led to resistance to trastuzumab (Pandya et al., 2016).

Targeting aberrant EGFR expression in cancer cells is a promising treatment strategy for NSCLC. Activation of the Hippo-YAP pathway was correlated with EGFR inhibitor treatment (Kim and Myung, 2018). Wang H et al. identified tankyrase and its associated E3 RNF146 as positive YAP activity regulators by CRISPR screening. Tankyrase inhibition by RNF146 enhanced the growth inhibitory activity of EGFR inhibitors in NSCLC by inhibiting YAP signaling (Wang et al., 2016). FBXW7 regulated quiescence by targeting the c-MYC protein for ubiquitination. High levels of FBXW7 and low levels of c-MYC were observed in gefitinib-resistant cells with EGFR exon 19 deletion, suggesting that FBXW7 plays a pivotal role in the maintenance of gefitinib resistance in EGFR mutation-positive NSCLC (Hidayat et al., 2019). Cetuximab is a monoclonal antibody with a molecular target of EGFR. Specific silencing of Cbl-b expression increased the expression of EGFR and decreased the sensitivity of GC cells to cetuximab (Yu et al., 2016).

Regorafenib and sorafenib are multikinase inhibitors of RAS/RAF/MEK/ERK signaling that function to prevent tumors. CRC cells containing FBW7-inactivating mutations, including missense mutations in three arginine residues (R465, R479, and R505), were found to be insensitive to regorafenib and sorafenib (Tong et al., 2017). Nanog is a master transcriptional regulator of stemness in CSCs. The E3 FBXW8 ubiquitinates Nanog and suppresses Nanog expression, resulting in stemness enhancement and sorafenib resistance (Cao et al., 2019).

JQ1 and I-BET, two selective inhibitors of the bromodomain and extraterminal (BET) family, have shown promising early clinical trial outcomes. Xiangpeng Dai et al. found that cullin-3SPOP was responsible for promoting BET protein degradation. PC cell lines derived from individuals harboring SPOP mutations had increased resistance to BET inhibitor-induced cell growth arrest and apoptosis (Dai et al., 2017). Moreover, specific SPOP mutations could impair BET degradation (Janouskova et al., 2017).

BRAF inhibitors (BRAFi) and MEK inhibitors (MEKi) provide rapid disease control in patients with BRAF-mutant metastatic melanoma (Kuske et al., 2018). BRAFi/MEKi resistance triggers proteasomal degradation of AMPK-α1 and consequently drives autophagy and metabolic reprogramming in melanoma cells. Li YY et al. discovered that RING finger 44 (RNF44) could earmark AMPK-α1 for ubiquitination-mediated degradation in BRAFi-resistant melanoma cells (Li et al., 2017). PARP inhibitors (PARPi) are used clinically to treat BRCA-mutated breast tumors. FBXO5 assembles the active SCF ubiquitin ligase complex constitutively targeting RAD51 for degradation. This mechanism controls BC biology and sensitivity to PARPi (Marzio et al., 2019). The E3s involved in the response to non-platinum anticancer drugs are listed in Table 1.

**TABLE 1:** Representative E3 ligase involved in non-platinum anti-cancer drug resistance.

Classification	Drug	Cancer	E3 ligase	Mechanism	Role	Ref
Plants	Taxol	NSCLC	FBXW7	FBXW7/MCL1/PLK1	Sensitive	Gasca et al. (2016)
		GC	FBXW7	MiR-363/FBXW7	Sensitive	Su et al. (2011)
		BC/OC/NPC	FBXW11	FBXW11/E1A/FOXO3	Resistance	Yang et al. (2016)
		NSCLC	Pirh2	Pirh2/p53	Resistance	Bian et al. (2020)
		BC	Parkin	Microtubule assembly/stabilization	Sensitive	Kim et al. (2015)
		Melanoma	SIAH2	miR-335/SIAH2/HDAC3	Resistance	Lukenbill and Kalaycio (2013)
	Docetaxel	PC	EDD	Wnt/β-Catenin	Resistance	Becker et al. (2008)
			SPOP	SPOP/Caprin	Sensitive	Wang et al. (2009)
	Camptothecin	CRC	HAUSP	p53/Mdm2	Sensitive	Shi et al. (2019)
Anti-metabolite	Fludarabine	HCC	COP1	COP1/p53-brn-3a/Bcl-2	Sensitive	Longley et al. (2003)
	5-Fluorouracil	BC	Skp2	Skp2/p27^Kip1^	Resistance	Kadera et al. (2015)
		PDACs	CBL	CBL/EGFR	Sensitive	Liang et al. (2019)
		CRC	TRIM47	TRIM47/SMAD4	Resistance	Fang et al. (2015)
			FBXW7	FBXW7/CRY2	Sensitive	Kalluri, (2016)
			RBCK1	Microenvironment/CAF	Resistance	Mini et al. (2006)
	Gemcitabine	PCC	FBXW7	FBW7/ENT1	Sensitive	Zhang et al. (2015)
			SMURF2	miR-15b/SMURF2/EMT	Sensitive	Yu et al. (2018)
			TRIM31	TRIM31/TRAF2/NF-κB	Sensitive	Hung et al. (2015)
		NSCLC	Cul4A	Cul4A/TGFβ1	Resistance	Asselin and Rizzari (2015)
	Asparaginase	Leukemias	FBXW7	Wnt pathway	Sensitive	Bhatt et al. (2017)
Alkylating	Temozolomide	GBM	HERC3	HERC3/SMAD7/TGFβ1	Resistance	Ko et al. (2018)
		GBM	MDM2	MDM2/NCL/HDAC	Resistance	Kamran et al. (2017)
Antibiotics	Doxorubicin	GC	FBXL7	AURKA/FBXL7/Survivin	Sensitive	Kamran et al. (2017)
		CRC	FBXO15	FBXO15/P-gp/mdr	Sensitive	Long et al. (2019)
		HCC	CUL2	CUL2/MAF-1	Resistance	Cheteh et al. (2020)
		HCC	FBXW7	FBXW7/HSF1/MDR1	Sensitive	Lin and Yamazaki, (2003)
		HCC	SIAH1	SIAH1/Zeb1/EMT	Resistance	Lee et al. (2016)
		Lymphoma	TRAF6	SMO/TRAF6	Resistance	Gelsomino et al., 2013)
		Colon cancer	Trc8	Trc8/HMGCoAR/MDR	Resistance	Xiao et al. (2017)
		Osteosarcoma	ZNRF2	miR-100/ZNRF2	Resistance	Qin et al. (2016)
		NSCLC	TRIM25	TRIM25/p53	Resistance	Wang et al. (2019)
		PC/BC	MDM2	FKBP12/MDM2/p53	Resistance	Xu et al. (2017)
	Adriamycin	BC/GC	Cbl-b	Cbl-b/EGFR/Akt-miR-200c-ZEB1 axis	Resistance	X CH et al. (2017); Zhang et al. (2015); Waks and Winer (2019)
Endocrine	Enzalutamide/abiraterone	PC	STUB1	STUB1/ar/ar-v7/HSP70	Resistance	Rulina et al. (2016)
		PC	Cullin-RING	AR–ERG/Wnt/β-catenin pathway/NF-κB pathway	Resistance	Li et al. (2017)
	Enzalutamide	PC	AMFR	11b-HSD2/cortisol	Resistance	Jordan (2003)
	Tamoxifen	BC	TRIM2	Bim/cleaved PARP/caspase 3	Resistance	Wang et al. (2017)
			HRD1	S100A8/HRD1	Sensitive	Donley et al. (2014)
			RBCK1	RBCK1/FKBPL/ERa	Resistance	Yin et al. (2019)
			FBXW2	FBXW2/Sox2	Sensitive	Yan et al. (2011)
			c-Cbl	c-Cbl/c-src/AKT	Resistance	Interiano et al. (2014)
			SIAH2	SIAH2/ER-a	Sensitive	Baccarani et al. (2019)
Targeted drugs	Imatinib	CML	c-Cbl	TGFβ/c-cbl/Lyn kinase activity	Resistance	Han et al. (2019)
			TRAF6	TRAF6/ULK1	Sensitive	Bigenzahn et al. (2018)
	Imatinib/rebastinib	CML	LZTR1	LZTR1/RAS/MAPK pathway	Sensitive	Scott et al. (2016)
	Bortezomib	MM	NEDD4-1	NEDD4-1/AKT	Sensitive	Hirosawa et al. (2018); Hirosawa et al. (2018)
			DTX3L	Cleaved PARP/caspase 3	Resistance	Malek et al. (2017)
			Skp2	Cullin-1/Commd1/caspase 3	Resistance	Huang et al. (2020)
	Lapatinib	BC/GC	c-Cbl/CHIP	HER2 degration	Sensitive	Nunes et al. (2016); Chan et al. (2012)
	Trastuzumab	BC	Skp2	AKT/Glut1/glucose uptake/glycolysis	Resistance	Kurokawa et al. (2013)
		GC	FBXW7	miR-223/FBXW7	Resistance	Pandya et al. (2016)
		BC	Mindbomb-1	Mindbomb-1/Jagged-1/Notch activation	Resistance	Kim and Myung (2018)
	Lapatinib	BC	MDM2	P53	Resistance	Eto et al. (2015)
	Erlotinib	NSCLC	RNF146	RNF146/Hippo-YAP pathway	Resistance	Hidayat et al. (2019)
	Gefitinib	NSCLC	FBXW7	FBXW7/c-MYC	Resistance	Yu et al. (2016)
	Cetuximab	GC	Cbl-b	Cbl-b/EGFR	Resistance	Tong et al. (2017)
	Sorafenib	HCC	FBXW8	Nanog/CSCs	Resistance	Dai et al. (2017)
	JQ1/I-BET	PC	Cullin-3SPOP	Cullin-3SPOP/BET	Resistance	Janouskova et al. (2017); Kuske et al. (2018)
	BRAFi/MEKi	Melanoma	RNF44	AMPK-α1/autophagy/metabolic	Resistance	Marzio et al. (2019)
	PARP inhibitors	BC	FBXO5	SCF complex/RAD51	Sensitive	Zhao et al. (2020)

## Discussion

### “Druggability” of E3s

The purpose of studying the mechanisms of resistance is to identify strategies to combat resistance. E3s control tumor drug resistance by specifically binding to drug resistance-related genes and controlling their expression. Therefore, targeting E3 ligases can serve as a potential and effective strategy for combatting drug resistance. In the current environment, studying the resistance mechanisms of all existing E3s is challenging and impractical. However, some E3s have shown their ability to combat resistance. Several kinds of E3s have been deemed “druggable”. First, E3s that target key pathway molecules, like Cbl, which targets EGFR, and NEDD4, which targets AKT, have potential (Zhao et al., 2020). Second, E3s targeting important oncogenes have also been proposed to be druggable. E3s like MDM2, which targets p53, FBXW2, which targets Sox2, and FBXW7, which targets MYC, have shown value in combating drug resistance.

### Targeting E3s to Combat Drug Resistance

Ongoing research mainly focuses on potential drugs that can be used in therapeutic applications targeting E3s. Such drugs must directly target the E3s to exert their effect; this requirement fits with several different tools, such as siRNAs, agonists, small molecule compounds that affect the binding of E3s to target proteins, and PROteolysis TArgeting Chimera (PROTAC) technology. Strategies targeting E3s include using small molecules or PROTACs that degrade E3s or interfere with the interaction between E3s and their substrates.

### Small Molecules Targeting E3s

**FIGURE 3:**
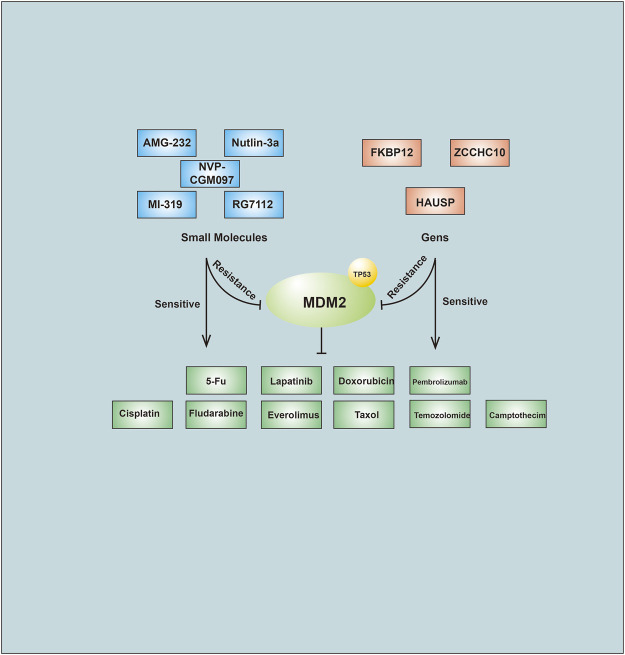
Small molecules compounds and Genes affect anti-cancer drug through regulation of MDM2.

Small molecules can target different E3s by directly binding with them and inhibiting their enzymatic activity. As mentioned above, MDM2 is a selective E3 that binds to P53. Agents that inhibit MDM2 include Nutlin-3a, RG7112, NVP-CGM097, AMG-232 and MI319. The combination of traditional anticancer drugs with novel agents was better for drug efficacy than monotherapy (Azmi et al., 1990; Kojima et al., 2006; Vu et al., 2013; Reuther et al., 2018) (Figure 3). These data suggest that targeting relevant E3s with small molecules to overcome drug resistance could be effective. Agonists have also been shown to be effective in controlling drug resistance. The PPARγ agonist pioglitazone inhibited EGFR/MDM2 signaling-mediated PPARγ degradation and increased cancer cell sensitivity to chemotherapy drugs (Shi et al., 2016).

### Interfering With the Interaction Between E3s and Their Substrates

Another strategy for combating drug resistance is interfering with the interaction between E3s and their target substrates. Using chemical library screens, E Malek et al. identified a novel compound, DT204, that reduced Skp2 binding to Cullin-1 and Commd1 and synergistically enhanced bortezomib-induced apoptosis (Malek et al., 2017). Small molecules have also been used to interfere with the interaction between EGFR and its E3. The downregulation of EGFR ubiquitination inhibits the internalization of EGFR, which is an essential mechanism of EGFR activation and drug resistance (Yu et al., 2020).

### Traditional Drugs Targeting E3s

Traditional drugs also facilitate the ubiquitination of E3s. Vitamin K3, an inhibitor of Siah2, promoted sensitivity of leukemia cells to imatinib (Huang et al., 2018). ATA is a molecular compound derived from Tanshinone IIA through chemical modification (Tian et al., 2010). A mechanistic study revealed that ATA promoted HER2 degradation by increasing c-Cbl and CHIP-mediated HER2 ubiquitination (Huang et al., 2020). Oridonin is a natural compound inducing oxidative stress that enhances CHIP targeting of BCR-ABL for ubiquitin-proteasome degradation, resulting in the enhancement of cancer cell death (Huang et al., 2017). Ginsenoside RD is another natural compound that increases the ubiquitination of multidrug resistance 1 (MDR1). Ginsenoside Rd treatment can reverse doxorubicin resistance in MCF-7/ADR cells (Pokharel et al., 2010).

### PROTACs That Degrade E3s

PROTAC technology utilizes the ubiquitin‐protease system to target a specific protein for ubiquitination and degradation (Sakamoto et al., 2001). PROTAC technology targets proteins, including transcription factors, skeletal proteins, enzymes, and regulatory factors (Zhang et al., 2018). Targeting oncogene family proteins using PROTACs to overcome drug resistance has recently become a popular area of research.

PROTACs directly targeting oncogenes are a tool for overcoming drug resistance. Ibrutinib resistance can occur due to a cysteine to serine mutation (C481S) in the site normally covalently bound by ibrutinib. Alexandru D. Buhimschi et al. introduced MT-802, which is a PROTAC that induces ubiquitination-dependent degradation of wild-type and C481S-mutant BTK from PROTAC, which could be a novel tool for overcoming ibrutinib resistance (Buhimschi et al., 2018). CP5V (apcin-A-PEG5-VHL ligand 1), as an efficient Cdc20 PROTAC, can mediate degradation of the oncogene Cdc20 through the ubiquitous pathway and overcome resistance to taxane chemotherapy in BC by inhibiting mitotic slippage (Chi et al., 2019). The SH2-U-box targets both native and T315I-mutant BCR-ABL for ubiquitination and degradation and thus may serve as a tool for treating both imatinib-sensitive and imatinib-resistant CML (Ru et al., 2016).

### Limitations of Targeting E3s to Combat Drug Resistance

In summary, E3s can play a role in tumor resistance by binding oncogenes or pathway proteins. Degrading E3s via different strategies or affecting their function can be used as strategies for anticancer treatment. However, E3s have some shortcomings in treating drug resistance. The first is the diversity of E3s, which prevents a complete understanding of all of the E3 functions and their corresponding target genes. The second is that E3s can bind to multiple different oncogenes. For example, TRIM family proteins perform different functions by binding to different oncogenes. The same E3 not only combines with one oncogene or tumor suppressor gene but also can interact with multiple oncogenes or tumor suppressor genes at the same time. Directly targeting E3s in anti-drug resistance research may lead to failure.

## Conclusion

Drug resistance has been a prominent factor negatively affecting clinical treatment. Various E3s target oncogenes or tumor suppressors, affecting the sensitivity of tumor cells to different drug treatments. Strategies that target E3s to combat drug resistance include interfering with E3 activators, degrading E3s and affecting the interaction between E3s and their substrates. Some famous E3s, like MDM2, FBWX7, and SKP2, have been well studied and shown to have value for treating drug resistance. Research on E3s has led to the discovery of possible therapeutic methods to overcome the challenging clinical situation imposed by drug resistance.
